# Association between gut microbiota and imaging biomarkers in arteriosclerotic cerebral small vessel disease with idiopathic normal pressure hydrocephalus

**DOI:** 10.3389/fnins.2026.1789687

**Published:** 2026-05-08

**Authors:** Jiaxin Cai, Kaiyan Feng, Jiaxin Chen, Lijuan Liang, Qiaoling Wu, Qishan He, Xiong Zhang, Jianming Lei, Jianhui Mai, Yizhong Li, Jinwen Feng, Chunnian Huang, Guofang Zeng, Chunwan Chen, Haifeng Huang, Shisheng Ye, Hao Li

**Affiliations:** 1 The First School of Clinical Medicine, Southern Medical University, Guangzhou, China; 2 Department of Neurology, Maoming People’s Hospital, Maoming, China; 3 The First School of Clinical College of Medicine, Guangdong Medical University, Zhanjiang, China; 4 Dongguan Chang'an Hospital, Dongguang, China; 5 Leliu Hospital Affiliated with Shunde Hospital of Guangzhou University of Chinese Medicine, Foshan, China; 6 Department of Integrated Therapy, Maoming People’s Hospital, Maoming, China; 7 Department of Radiology, Maoming People’s Hospital, Maoming, China; 8 Jinan University, Guangzhou, China

**Keywords:** arteriosclerotic cerebral small vessel disease, gut microbiota, idiopathic normal pressure hydrocephalus, imaging biomarker, Solobacterium

## Abstract

**Objectives:**

This study aims to characterize the gut microbiota in arteriosclerotic cerebral small vessel disease and idiopathic normal pressure hydrocephalus (aCSVD-iNPH) patients, investigate its correlation with cerebral small vessel disease imaging markers, and develop a diagnostic model based on differential microbial profiles.

**Methods:**

This study enrolled 95 subjects. Fresh fecal samples were collected for microbial DNA extraction. Gut microbiota composition was analyzed using 16S rDNA sequencing, and Spearman’s correlation analysis was performed to investigate the association between microbial abundance and imaging indicators.

**Results:**

An analysis of gut microbiota diversity revealed no statistically significant differences in *α*-diversity among the three groups, but there were significant differences in *β*-diversity. Compared to the aCSVD group, the relative abundances of Clostridium_Innocuum_group, Dietzia, Lachnospira, Pedobacter, Romboutsia, Saccharomyces, Solobacterium, Thermus, and TM7x were significantly higher in the aCSVD-iNPH group (all *p* < 0.05). Among these, the relative abundance of Solobacterium was negatively correlated with the number of cerebral microbleeds (CMBs) in the basal ganglia (*r* = −0.292, FDR-adjusted *p* = 0.019) and deep regions (*r* = −0.296, FDR-adjusted *p* = 0.018). Additionally, the relative abundance of Thermus was positively correlated with the deep medullary vein (DMV) score (*r* = 0.271, FDR-adjusted *p* = 0.031). The ROC analysis yielded AUC values ranging from 0.666 to 0.716, indicating limited discriminative ability between aCSVD-iNPH and aCSVD (Thermus, AUC = 0.716, 95% CI 0.569–0.864, *p* = 0.006; Saccharomyces, AUC = 0.688, 95% CI 0.535–0.842, *p* = 0.017; Dietzia, AUC = 0.666, 95% CI 0.512–0.821, *p* = 0.034).

**Conclusion:**

This study identifies distinct changes in gut microbiota among patients with aCSVD-iNPH and their associations with aCSVD imaging markers, suggesting that certain microbial taxa may serve as potential diagnostic biomarkers to distinguish aCSVD-iNPH from aCSVD, although these preliminary findings require validation in independent cohorts.

## 1 Introduction

Cerebral small vessel disease (CSVD) encompasses a range of conditions that affect the brain’s small arteries, arterioles, capillaries, and venules, ultimately leading to brain tissue injury (Pantoni, 2010; Mishra et al., 2019). It is predominantly found in individuals over the age of 60 and is frequently linked to small artery sclerosis or cerebral amyloid angiopathy, collectively termed age-related CSVD (ArCSVD). This condition involves the degeneration of cerebral small vessels and primarily manifests in two forms: arteriosclerotic CSVD (aCSVD) and cerebral amyloid angiopathy (Lu and Song, 2019).

As a key subtype of ArCSVD, aCSVD exhibits a high prevalence in the elderly. Its pathogenesis is influenced by multiple factors, including endothelial dysfunction, inflammatory responses, and genetic predispositions (Huang J. et al., 2024). aCSVD principally impacts small arteries, veins, and deep perforating vessels originating from the skull base (Pantoni, 2010). Pathological hallmarks include small artery sclerosis, fibrinoid necrosis, and lipohyalinosis, characterized by endothelial proliferation, media degeneration, and vascular wall thickening. Endothelial injury and blood–brain barrier (BBB) dysfunction are central drivers of this pathology (Grinberg and Thal, 2010). Given its strong associations with aging and vascular risk factors such as hypertension, aCSVD is often labeled as age-related or vascular risk factor-related CSVD (Pantoni, 2010; Banerjee et al., 2016). Notably, deep microbleeds are typically associated with arteriosclerotic vasculopathy, whereas lobar microbleeds are more often linked to cerebral amyloid angiopathy (Das et al., 2023). Emerging evidence suggests that gut microbiota may play a potential role in CSVD, possibly by influencing the host’s immune system, metabolism, and neuroendocrine pathways (Cai et al., 2021).

Normal pressure hydrocephalus (NPH) is a clinical syndrome characterized by the triad of gait instability, cognitive impairment, and urinary incontinence, which typically progresses gradually. Radiologically, it presents with ventricular enlargement despite normal cerebrospinal fluid (CSF) pressure (70–200 mmH₂O). NPH is classified as secondary (resulting from known causes such as head trauma or infection) or idiopathic (iNPH), which is a neurological disorder mainly affecting the elderly. The pathogenesis of iNPH is complex and shows clinical overlap with CSVD; many patients experience improvement following CSF shunt surgery (Klinge et al., 2012). While the classic triad is present in approximately half of iNPH patients (Shoamanesh et al., 2011), other symptoms such as headache, dizziness, and parkinsonism may occur. Early surgical intervention significantly enhances prognosis. Notably, iNPH patients often exhibit CSVD radiological markers, and CSVD severity may influence surgical outcomes (Roth et al., 2020; Cai et al., 2025). Crucially, BBB impairment is a shared pathological feature of both CSVD and iNPH, and gut microbiota dysbiosis is hypothesized to contribute to both diseases via BBB disruption (Roth et al., 2020; Cai et al., 2025).

Key neuroimaging features of CSVD include white matter hyperintensities (WMHs), cerebral microbleeds (CMBs), enlarged perivascular spaces (EPVSs), lacunes, and abnormalities of the deep medullary veins (DMVs) (Zhao et al., 2020). The DMVs are essential for maintaining normal white matter perfusion. Disruptions such as venous discontinuity, occlusive collagenosis, or reduced visibility on high-resolution susceptibility-weighted imaging have been implicated in CSVD pathogenesis and are spatially associated with periventricular and focal WMHs (Zhang et al., 2025). Accumulating evidence suggests that DMV abnormalities correlate with multidimensional cognitive impairment and may reflect underlying venous dysfunction that contributes to white matter injury (Zhang et al., 2025). Collectively, these imaging markers reflect the CSVD burden and are associated with cognitive decline (Rahmani et al., 2022; Tap et al., 2023). Nevertheless, research investigating the relationship between gut microbiota and CSVD imaging markers remains limited, particularly in patients with coexisting aCSVD-iNPH.

The co-occurrence of aCSVD and iNPH is clinically observed in older adults; however, its underlying pathophysiological mechanisms are unclear. The gut–brain axis, which is a complex bidirectional communication network, links gastrointestinal function to brain health. Gut microbiota is implicated in various central nervous system disorders, including Parkinson’s disease, multiple sclerosis, and depression (Tap et al., 2023; Yu and Zhao, 2021; Robinson et al., 2024; Doifode et al., 2021), highlighting its potential as a biomarker and therapeutic target. Studies indicate that gut microbiota may influence aCSVD pathogenesis through immune regulation, microbial metabolites, and systemic inflammation (Cai et al., 2021). Nevertheless, the characteristics of gut microbiota in aCSVD-iNPH patients and their relationship with CSVD imaging markers remain largely unexplored.

This study aims to define the gut microbiota profile in aCSVD-iNPH patients and examine its correlation with CSVD neuroimaging markers. By comparing the gut microbial community structures in aCSVD-iNPH patients, aCSVD-only patients, and healthy controls, we seek to elucidate potential links between gut microbiota and the severity of CSVD as indicated by imaging. We hope that these findings will clarify the role of gut microbiota in the pathophysiology of aCSVD-iNPH and provide a preliminary foundation for future microbiota-based diagnostic and therapeutic approaches.

## 2 Materials and methods

### 2.1 Study population

This study is a single-center cross-sectional study. From August 2020 to November 2024, a total of 20 patients with aCSVD comorbid with iNPH, 45 patients with aCSVD alone, and 30 elderly healthy control (HC) subjects undergoing routine physical examinations at the health check-up center were consecutively recruited, screened, and enrolled from the inpatients and outpatients of the Department of Neurology in our hospital.

#### 2.1.1 Inclusion of aCSVD

The diagnostic criteria referred to “the Chinese Expert Consensus on the Diagnosis and Treatment of Cerebral Small Vessel Disease” (Writing Group of the 2021 Chinese Expert Consensus on Diagnosis and Treatment of Cerebral Small Vessel Disease, Professional Committee on Cerebral Small Vessel Disease, Chinese Research Hospital Association, 2021). Inclusion criteria included: (1) age of 60 years or older; (2) presence of at least one of the following atherosclerotic risk factors: hyperhomocysteinemia, smoking, alcohol consumption, body mass index (BMI) > 25, hypertension, diabetes mellitus, coronary heart disease, or dyslipidemia; and (3) neuroimaging findings on magnetic resonance imaging (MRI) meet the STRIVE recommended criteria (Wardlaw et al., 2013). All included patients presented with deep microbleeds, including those in the brainstem, dentate nucleus, and basal ganglia, regardless of cerebellar or lobar microbleeds (Das et al., 2023). (4) No history of traumatic brain injury or intracranial space-occupying lesions; (5) No central nervous system lesions secondary to infection, metabolic disorders, immune diseases, poisoning, or malignancies; (6) No ischemic stroke caused by cerebral arterial occlusion or cardioembolic events; (7) No severe atherosclerotic stenosis of the cerebral arteries that may alter cerebral hemodynamics; (8) No cerebral hemorrhage.

#### 2.1.2 Inclusion of iNPH

The diagnostic criteria referred to the 2012 International Guidelines for iNPH (Klinge et al., 2012; Ishikawa et al., 2008). Inclusion criteria included: (1) age of 60 years or older; (2) presence of at least one of the following clinical triad symptoms: gait disturbance, cognitive impairment, and urinary incontinence; (3) ventricular dilation (Evans’ index > 0.3); (4) cerebrospinal fluid (CSF) pressure of 180 mmHg or lower; (5) presence of at least one of the following three research features: ① neuroimaging findings of narrowed sulci and subarachnoid spaces over the cerebral convexity/midline surface in the presence of gait disturbance and ② improvement of symptoms after a cerebrospinal fluid drainage test. (6) The above clinical symptoms cannot be fully explained by other neurological or non-neurological diseases. (7) No diseases that may cause ventricular dilation, including subarachnoid hemorrhage, meningitis, head trauma, congenital hydrocephalus, and aqueductal stenosis.

#### 2.1.3 Inclusion of healthy groups

Inclusion criteria included: (1) healthy individuals aged 60 years or older; (2) no history of cerebral infarction, cerebral hemorrhage, anxiety, depression, encephalitis, angina pectoris, or myocardial infarction; (3) no history of cancer; (4) no history of dementia; (5) no vascular risk factors such as smoking, hypertension, diabetes mellitus, or dyslipidemia; (6) no significant abnormalities found on cranial magnetic resonance imaging (MRI); and (7) individuals who do not meet the inclusion criteria for aCSVD and iNPH in this study.

#### 2.1.4 Grouping of participants

This study established three distinct groups: (1) aCSVD Group: participants meeting the diagnostic criteria outlined in Section 2.1.1; (2) aCSVD-iNPH Group: participants fulfilling both the diagnostic criteria specified in Sections 2.1.1 and 2.1.2; (3) healthy control (HC) group: participants satisfying the selection criteria described in Section 2.1.3.

### 2.2 Collection of clinical data

Collect the following clinical data: (1) demographic information, including the patient’s gender and age; (2) medical history, including history of ischemic stroke, smoking, hypertension, and diabetes; (3) laboratory indicators, including levels of homocysteine (HCY), low-density lipoprotein cholesterol (LDL-C), triglycerides, and cholesterol; (4) imaging data, mainly magnetic resonance imaging (MRI) examinations, covering sequences such as brain MRI plain scan, susceptibility-weighted imaging (SWI), and fluid-attenuated inversion recovery (FLAIR); (5) gut microbiota indicators, including the richness, diversity, and structural composition of the microbial community.

### 2.3 Imaging analysis of DMVs

Based on the anatomical characteristics of DMVs’ distribution, the levels with the highest DMV visualization on clinical brain susceptibility-weighted imaging (SWI) sequences are primarily located in the centrum semiovale and basal ganglia regions, particularly at the midbody level of the lateral ventricles. This study consecutively selected five sections, from the base of the centrum semiovale to the point where the bilateral lateral ventricles converge into the third ventricle. The analysis method referenced previously reported approaches for DMVs’ assessment in CSVD (Chen et al., 2020). Two experienced neuroradiologists, who were blinded to all clinical and grouping information, independently evaluated the DMVs’ scores based on the previously described method. Any discrepancies were resolved by consensus with a third senior neuroradiologist.

### 2.4 Experimental methods for gut microbiota detection

The gut microbiota was analyzed by extracting total DNA from fecal samples using standard protocols. DNA concentration and quality were assessed by spectrophotometry and agarose gel electrophoresis. The V3–V4 region of the 16S rRNA gene was amplified using Pfu high-fidelity DNA polymerase with barcoded primers; negative controls were included to monitor contamination, and any batch showing amplification in controls was excluded. PCR products were purified using magnetic beads, quantified with the Quant-iT PicoGreen dsDNA Assay Kit, and pooled accordingly.

Libraries were constructed using the Illumina TruSeq Nano DNA LT Library Prep Kit, involving end repair, A-tailing, adapter ligation with unique indexes, magnetic bead purification, PCR enrichment, and size selection by gel electrophoresis. Library quality and concentration were assessed using an Agilent Bioanalyzer and fluorometric quantification, with qualified libraries exceeding 2 nM. Paired-end sequencing was performed on an Illumina MiSeq or NovaSeq 6,000 platform using appropriate reagent kits, generating reads for subsequent bioinformatics analysis of microbial diversity.

### 2.5 Statistical analysis

**Figure 1:**
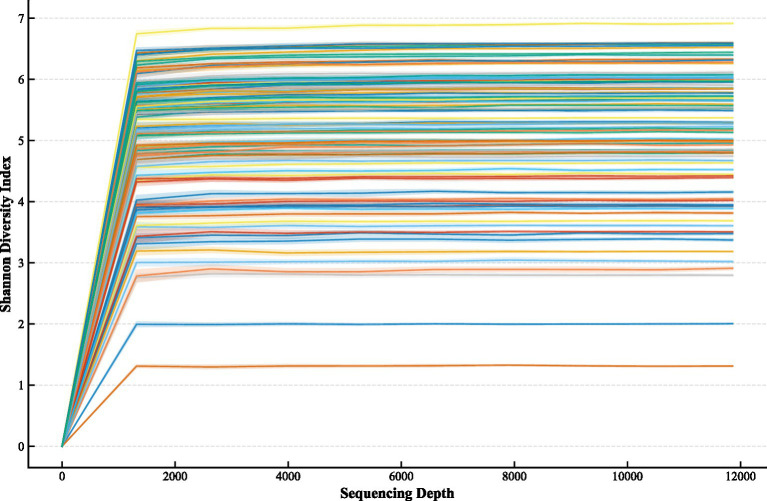
Rarefaction curve.

Statistical analyses were performed using SPSS 25.0. Data normality was assessed using the Shapiro–Wilk test. Continuous variables are presented as mean ± standard deviation or median (interquartile range), while categorical variables are expressed as frequency (percentage). Group comparisons were conducted using an independent samples t-test (normal distribution), a Mann–Whitney U-test (non-normal distribution), χ^2^ test, or Fisher’s exact test, as appropriate. For multiple pairwise comparisons among the three groups, the Benjamini–Hochberg false discovery rate (FDR) correction was applied (Figure 1).

Spearman correlation analysis was used to examine associations between gut microbiota OTUs and radiological markers (lacune count, white matter hyperintensity score, microbleed count, and DMV score). Receiver operating characteristic (ROC) curve analysis was performed to evaluate the diagnostic potential of genus-level gut microbiota abundance changes for aCSVD-iNPH.

**Figure 2:**
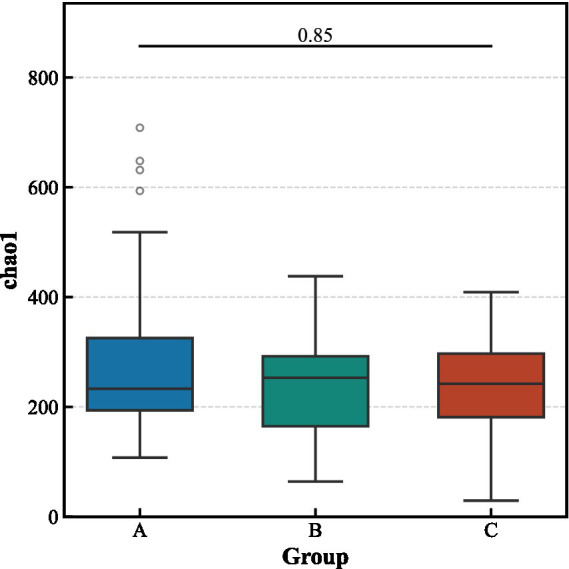
Venn diagram. **(A)** aCSVD group; **(B)** aCSVD‑iNPH group; **(C)** HC group.

Microbial diversity was analyzed by calculating *α*-diversity indices (Shannon, Simpson, Chao1, Observed OTUs, and Pielou’s evenness) and *β*-diversity matrices (Jaccard and Bray–Curtis). Inter-group differences were tested using PERMANOVA with principal coordinate analysis (PCoA) visualization. Genus-level abundance differences were assessed using the Kruskal–Wallis H test, and signature species were identified through LEfSe analysis (LDA > 2.0, *p* < 0.05). Finally, Spearman’s correlation analysis was reapplied to clarify relationships between specific OTUs and radiological markers in aCSVD-iNPH patients (Figure 2).

## 3 Result

### 3.1 Baseline characteristics and radiological markers

A total of 95 subjects were enrolled in this study, including 45 in the aCSVD group, 20 in the aCSVD with aCSVD-iNPH group, and 30 in the healthy control (HC) group. No significant differences were observed in baseline characteristics such as gender, age, or triglyceride levels among the three groups. However, serum total cholesterol levels were significantly lower in the aCSVD-iNPH group compared to the aCSVD group (4.45 vs. 5.13, *p* < 0.05). Regarding radiological markers, the aCSVD-iNPH group demonstrated significantly higher deep WMH Fazekas scores (*p* < 0.05) and DMV scores (*p* < 0.001) than the aCSVD group, while no significant differences were found in other imaging markers, including lacunar infarct counts and CMBs (Table 1).

**Table 1:** Demographic and clinical parameters among the three groups.

Variables	aCSVD (*n* = 45)	aCSVD-iNPH (*n* = 20)	HC (*n* = 30)
Age	67.40 ± 8.85	70.50 ± 7.82	66.77 ± 5.92
Male (%)	32 (71. 10%)	17 (85.00%)	18 (60.00%)
History of hypertension (%)	39 (86.70%)	15 (75.00%)	-
History of diabetes (%)	11 (24.40%)	6 (30.00%)	-
History of smoking (%)	2 (4.40%)	1 (5.00%)	-
Previous stroke (%)	33 (73.30%)	14 (70.00%)	-
Triglycerides (mmol/L)	1.41 (0.98,2. 15)	1.1 7 (0.86, 1.98)	1.20 (0.93, 1.77)
Cholesterol (mmol/L)	5. 13 ± 1.17^**^	4.45 ± 0.84^#**^	4.07 ± 0.79
LDL-C (mmol/L)	2.97 (2.26,3.52)^**^	2.73 (1.92,3.15)^**^	2.07 (1.69,2.40)
HCY (mmol/L)	18.27 ± 8.00^*^	18.68 ± 5.37^*^	12.60 ± 2.83
Lacune, *n*	5.09 ± 4.16	5.05 ± 3.97	-
Periventricular WMH Fazekas	2.37 ± 0.73	1.75 ± 1.02	-
Deep WMH Fazekas	1.65 ± 0.72	1.80 ± 1.06^#^	-
Brainstem CMBs, *n*	3.98 ± 6.15	3.00 ± 4.03	-
Dentate Nucleus CMBs, *n*	1.64 ± 2.68	2.40 ± 3.35	-
Cerebellum CMBs, *n*	1.69 ± 3.92	1.20 ± 1.77	-
BG CMBs, *n*	13.31 ± 10.09	14.60 ± 13.22	-
Deep CMBs, *n*	18.93 ± 16.51	20.00 ± 17.93	-
Lobe CMBs, *n*	10.02 ± 11.47	14.70 ± 15.81	-
Total CMBs, *n*	28.95 ± 25.56	34.70 ± 32.75	-
EPVS-BG-L Scores	2.37 ± 1.02	2.05 ± 1.10	-
EPVS-BG-R Scores	2.23 ± 1.13	2.20 ± 1.28	-
DMV scores	11.50 ± 2.64	16.13 ± 2.66*^##^*	-
Burden Scores	3.51 ± 0.60	3.55 ± 0.69	-

^*^Compared with HC, ^*^p < 0.05, ^**^p < 0.001; #compared with aCSVD, ^#^*p* < 0.05, ^##^*p* < 0.001; LDL-C, low-density lipoprotein cholesterol; HCY, homocysteine; WMHs, white matter hyperintensities; CMBs, cerebral microbleeds; EPVSs, enlarged perivascular spaces; BG, basal ganglia; DMVs, deep medullary veins; Burden score, cerebral small vessel disease burden score.

### 3.2 Analysis of gut microbiota diversity

Figure 1 presents the rarefaction curves, indicating that the sequencing depth was sufficient to capture the microbial diversity across all samples. Figure 2 shows the Venn diagram illustrating the number of shared and unique OTUs among the three groups.

**Figure 3:**
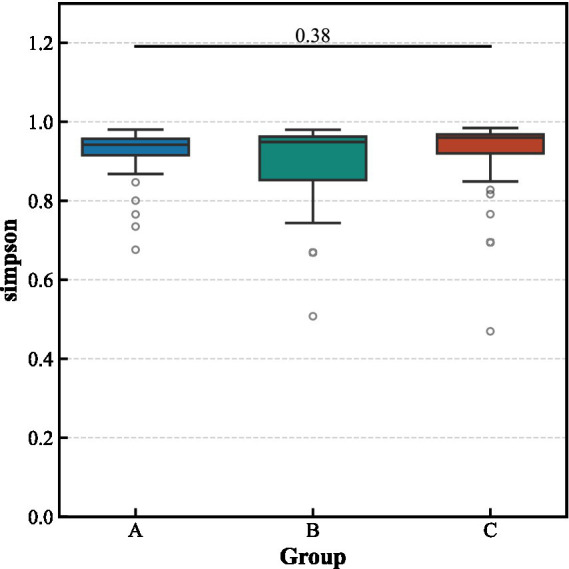
Alpha-diversity indices among the three groups.

**Figure 4:**
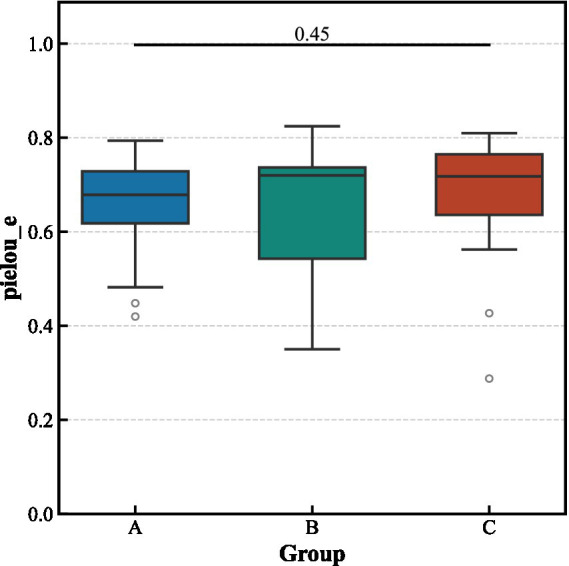
Beta-diversity indices among the three groups.

**Figure 5:**
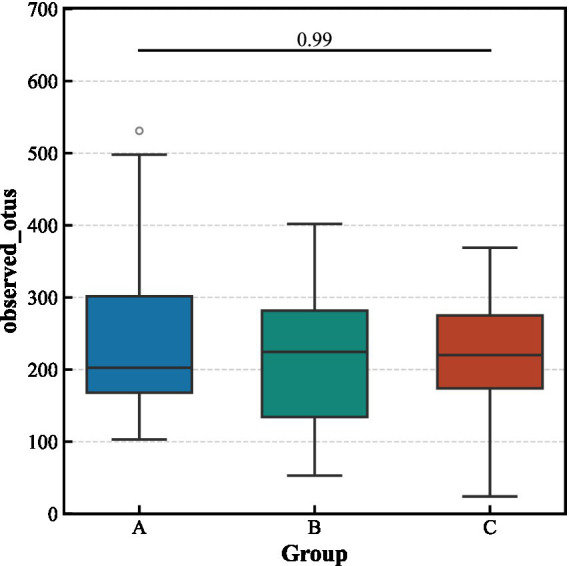
Beta-diversity indices among the three groups.

Alpha diversity analysis revealed no significant differences in the richness and evenness of the gut microbiota among the three groups (Figure 3). In contrast, beta diversity analysis indicated significant structural separation of the microbial communities (Bray–Curtis distance, pseudo-*F* = 1.463, *p* < 0.001). Notably, the differences between the aCSVD-iNPH and aCSVD groups were primarily manifested in the presence or absence of species composition (Jaccard distance, *p* = 0.013), rather than in relative abundance (Bray–Curtis distance, *p* > 0.05), suggesting that the disease status leads to the colonization or absence of specific bacterial taxa (Figures 4, 5).

### 3.3 Identification of intergroup differential taxa

**Figure 6:**
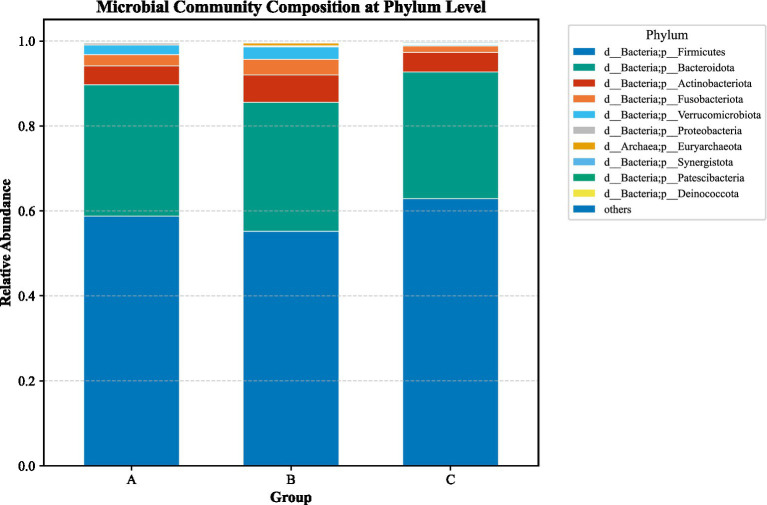
Phylum-level relative abundances track plot among three groups. **(A)** ACSVDgroup; **(B)** ACSVD-iNPH group; **(C)** HC group.

**Figure 7:**
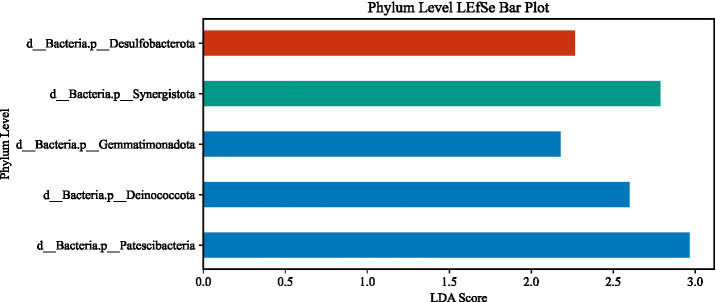
Phylum-level LEfSe analysis. **(A)** aCSVD group; **(B)** aCSVD-iNPH group; **(C)** HC group.

**Figure 8:**
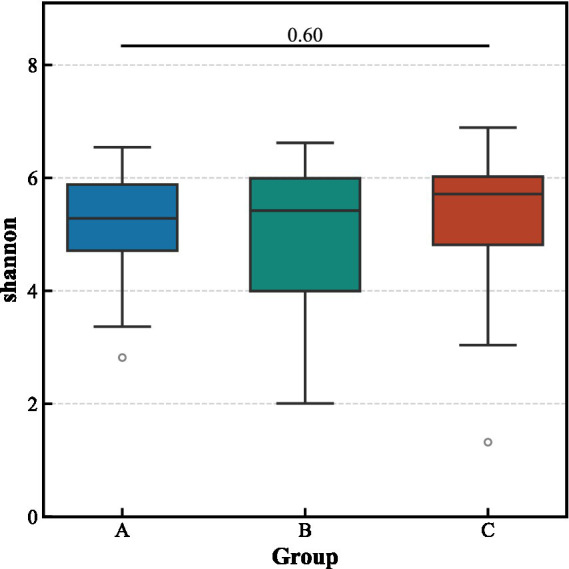
Class-level LEfSe analysis. **(A)** aCSVD group; **(B)** aCSVD-iNPH group; **(C)** HC group.

**Figure 9:**
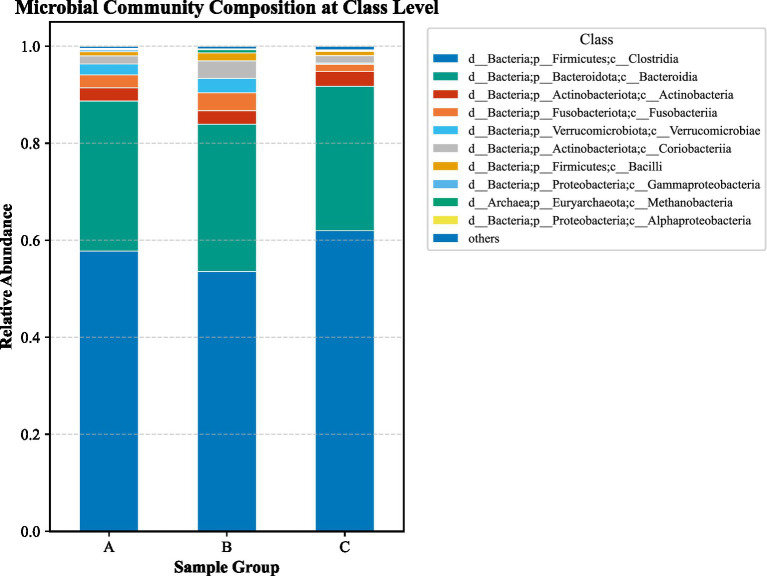
Class-level relative abundance stacked plot among three groups. **(A)** aCSVD group; **(B)** aCSVD-iNPH group; **(C)** HC group.

**Figure 10:**
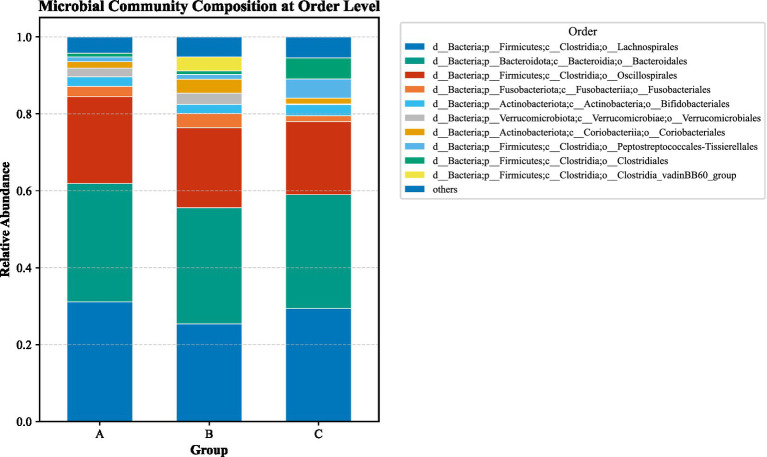
Order-level relative abundance stacked plot among three groups. **(A)** aCSVD group; **(B)** aCSVD-iNPH group; **(C)** HC group.

**Figure 11:**
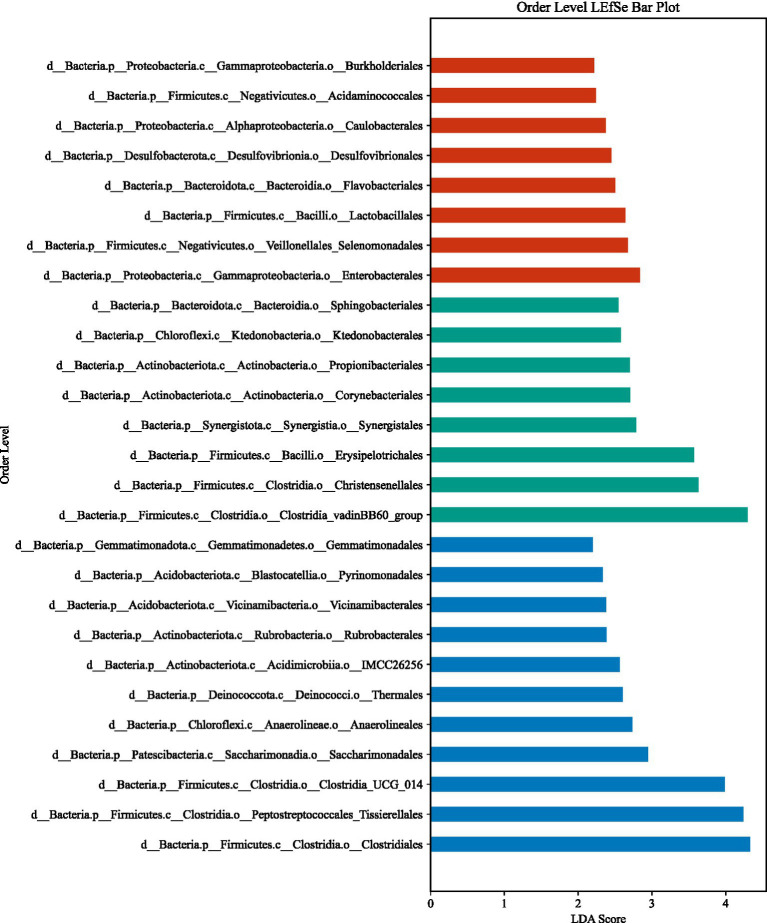
Order-level LEfSe analysis. **(A)** aCSVD group; **(B)** aCSVD-iNPH group; **(C)** HC group.

**Figure 12:**
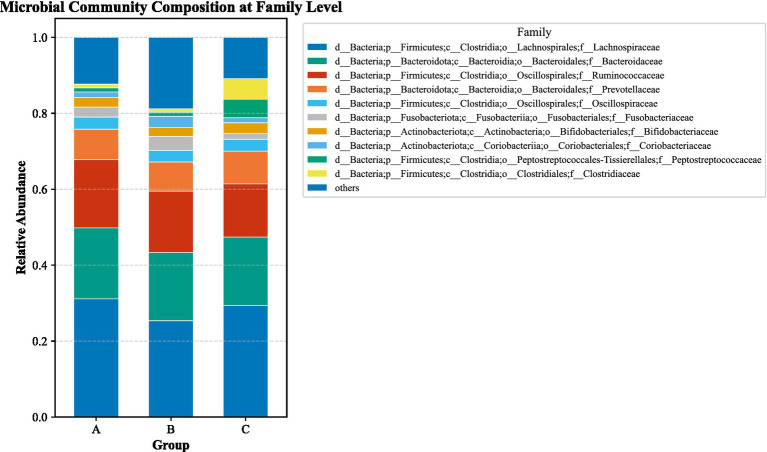
Family-level relative abundance stacked plot among three groups. **(A)** aCSVD group; **(B)** aCSVD-iNPH group; **(C)** HC group.

**Figure 13:**
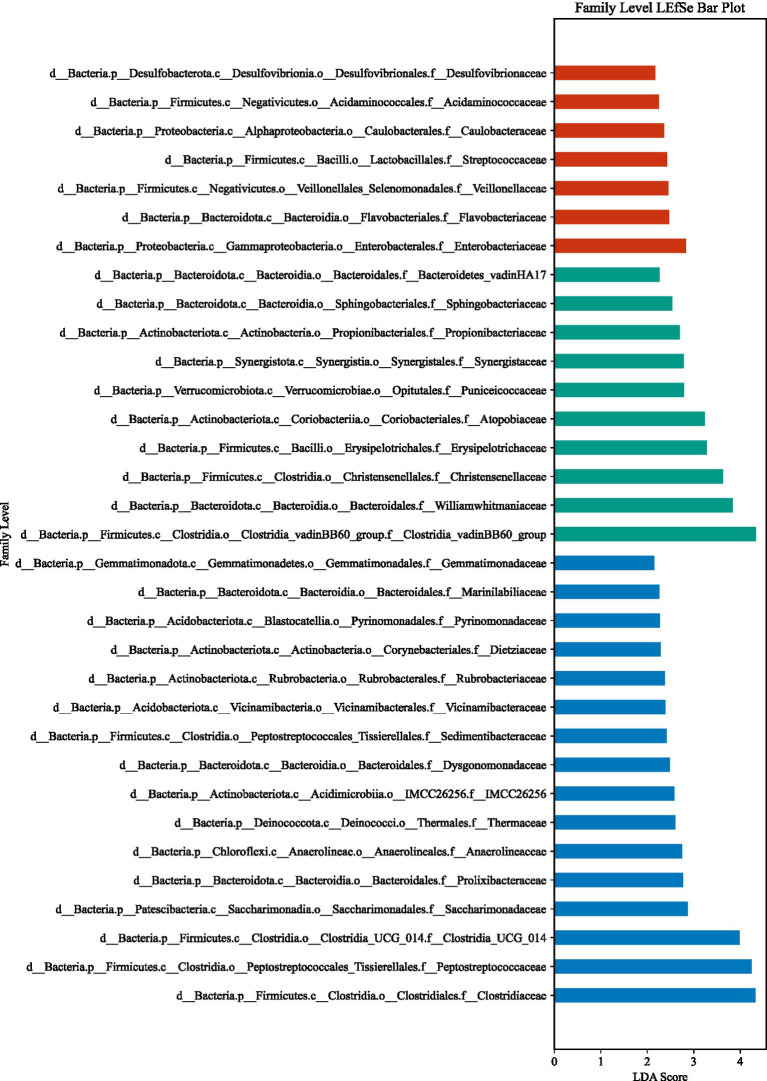
Family-level LEfSe analysis. **(A)** aCSVD group; **(B)** aCSVD-iNPH group; **(C)** HC group.

LEfSe analysis (LDA > 2, *p* < 0.05) identified substantial differences in microbial taxa across groups at the phylum to family levels (Figures 6–13). Complete lists of differentially abundant taxa at each taxonomic level are provided in the Supplementary materials. Based on these findings, subsequent in-depth analysis was focused on the genus level due to its greater biological specificity.

### 3.4 Identification of characteristic genera, imaging correlations, and diagnostic model development in aCSVD

**Figure 14:**
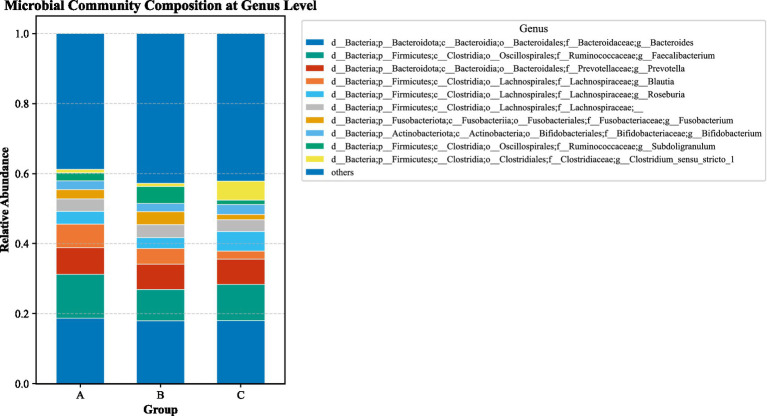
Genus-level relative abundance stacked plot among three groups. **(A)** aCSVD group; **(B)** aCSVD-iNPH group; **(C)** HC group.

**Figure 15:**
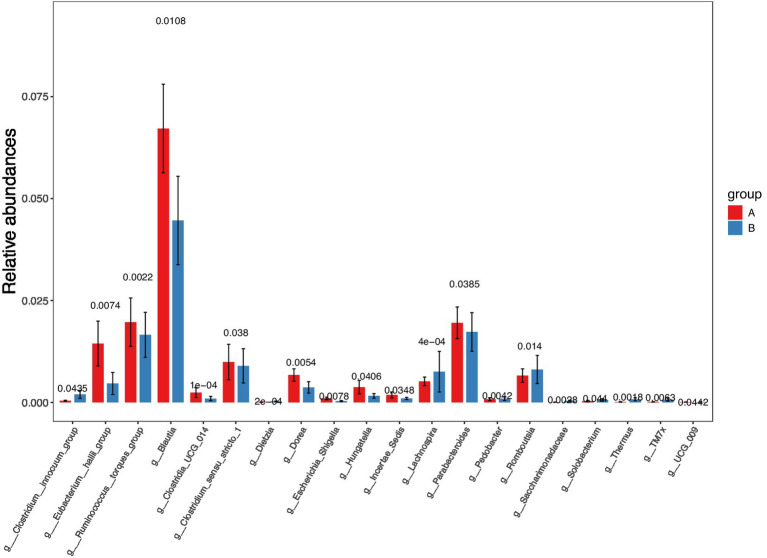
Genus-level LEfSe relative abundance among two groups. **(A)** aCSVD group; **(B)** aCSVD-iNPH group.

**Figure 16:**
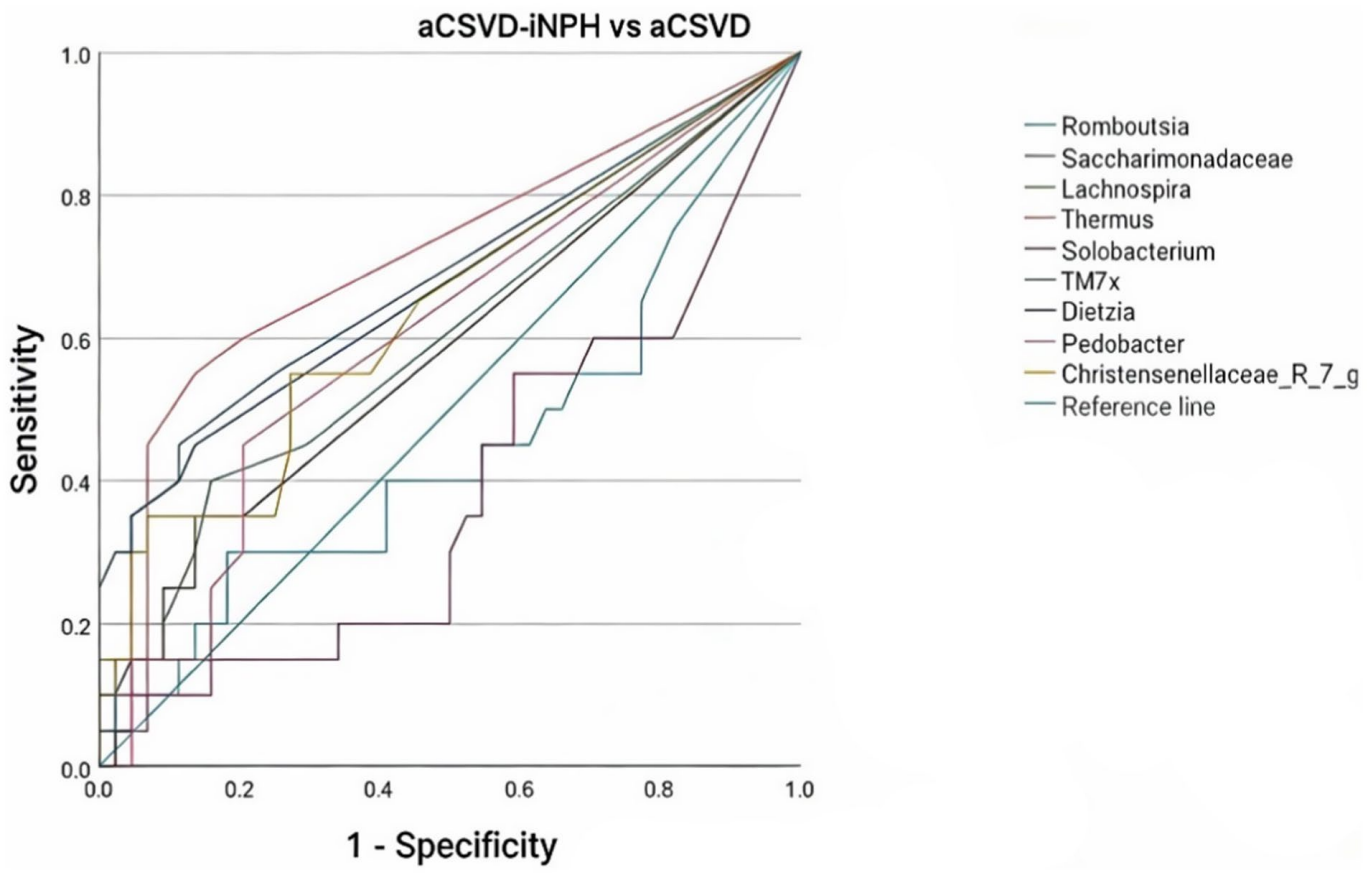
ROC curve between aCSVD-iNPH and aCSVD.

Analysis at the genus level revealed that, compared to the aCSVD group, the aCSVD-iNPH group exhibited significant enrichment of 9 bacterial genera (including Clostridium_innocuum_group, Dietzia, and Thermus), while 11 genera were significantly depleted (Figure 14; see Supplementary Table S1 for the complete list). We focused specifically on those genera showing significant correlations with cerebral small vessel disease imaging markers. Spearman correlation analysis was performed between genus-level intestinal microbial abundance and neuroimaging markers. Solobacterium abundance showed a significant inverse correlation with both basal-ganglia CMBs (*r* = −0.292, FDR-adjusted *p* = 0.019) and deep CMBs (*r* = −0.296, FDR-adjusted *p* = 0.018). Conversely, Thermus abundance exhibited a positive correlation with DMV score (*r* = 0.271, FDR-adjusted *p* = 0.031) (Figure 15). Based on these differentially abundant genera, we further constructed a discriminant model. The ROC analysis yielded AUC values ranging from 0.666 to 0.716, reflecting a limited level of diagnostic accuracy between aCSVD-iNPH and aCSVD (Thermus, AUC = 0.716, 95% CI 0.569–0.864, *p* = 0.006; Saccharomyces, AUC = 0.688, 95% CI 0.535–0.842, *p* = 0.017; Dietzia, AUC = 0.666, 95% CI 0.512–0.821, *p* = 0.034) (Figure 16).

## 4 Discussion

This study represents the first investigation to analyze the gut microbiota structure in patients with aCSVD comorbid with iNPH and its correlation with imaging markers. aCSVD is a common subtype of cerebral small vessel disease, characterized by an insidious onset and a rising prevalence with population aging. aCSVD and iNPH share common risk factors (Deng et al., 2023; Javierre-Petit et al., 2024; Switzer et al., 2024; Zou et al., 2022) and imaging markers (Roth et al., 2020; Switzer et al., 2024; Jeppsson et al., 2022; Park et al., 2019; Xia et al., 2020), suggesting a potential overlapping pathogenesis (Roth et al., 2020; Jeppsson et al., 2022). Clinically, the comorbidity of aCSVD and iNPH is not rare. Such patients often exhibit high dependency in daily living and are prone to repeated hospitalizations, yet they are frequently overlooked by neurologists and are generally classified as ordinary stroke or dementia cases, neglecting specialized diagnosis and treatment for iNPH.

Previous studies have indicated that gut microbiota participate in the pathogenesis of aCSVD through immune regulation, metabolic pathways, and systemic inflammation. Patients with aCSVD show reduced microbial diversity, and the abundance of specific bacterial genera is associated with arteriosclerosis (Huang J. et al., 2024; Das et al., 2023). iNPH often co-occurs with aCSVD, and its imaging markers (e.g., WMHs) can exacerbate cognitive and gait impairments (Roth et al., 2020). However, whether the comorbidity of aCSVD and iNPH is influenced by gut microbiota remains unexplored. This study compared the diversity and composition of gut microbiota among the aCSVD-iNPH group, aCSVD group, and healthy controls (HC group) and conducted a preliminary analysis of the correlation between gut microbiota and imaging markers of cerebral small vessel disease.

This study found no significant differences in the alpha diversity of gut microbiota among the three groups, but significant distinctions in beta diversity were observed between the aCSVD-iNPH group, the aCSVD group, and the HC group. This suggests that disease states primarily participate in the pathological process by altering the microbial community structure rather than overall diversity. These findings align with previous research indicating that gut microbiota dysbiosis exists in cerebral small vessel disease and influences disease progression through immune and metabolic pathways (Huang J. et al., 2024; Das et al., 2023). Notably, the differences in beta diversity between the aCSVD-iNPH group and the aCSVD group were mainly reflected in the presence or absence of specific bacterial genera, rather than differences in their abundance. This indicates that the comorbidity of aCSVD and iNPH may involve the selective enrichment or suppression of particular bacterial taxa, thereby exacerbating small vessel damage and potentially promoting the progression from aCSVD to a comorbid iNPH state.

Analysis of general clinical data revealed that the aCSVD-iNPH group had significantly lower serum cholesterol levels compared to the aCSVD group, which may be associated with gut microbiota-mediated metabolic regulation. This study found significant enrichment of the *Clostridium innocuum* group and Lachnospira in the aCSVD-iNPH group, while butyrate-producing bacteria such as the *Eubacterium hallii* group showed reduced abundance. Research indicates that Clostridium species may promote cholesterol excretion through the production of coprostanol, thereby reducing serum cholesterol levels (Liu et al., 2025; Kenny et al., 2020). Concurrently, the reduction in bacteria such as the *Eubacterium hallii* group may weaken the inhibitory effect of short-chain fatty acids on hepatic HMG-CoA reductase, leading to restricted compensatory enhancement of endogenous cholesterol synthesis (Kim and Song, 2020; Harshfield and Markus, 2023). This “bidirectional regulatory imbalance” (increased excretion and constrained synthesis) could be one potential mechanism underlying observed hypocholesterolemia. Furthermore, the comorbidity of iNPH with aCSVD may exacerbate blood–brain barrier (BBB) dysfunction. Studies have detected extravasated plasma lipoproteins in the cerebrospinal fluid of iNPH patients, suggesting that BBB leakage may lead to central-peripheral cholesterol redistribution (Escudero-Martinez et al., 2023; Tseng et al., 2024). Cholesterol is a key component of maintaining BBB integrity. Its downregulation may reduce membrane fluidity in cerebral microvascular endothelial cells, further exacerbating BBB leakage and creating a vicious cycle (Rodrigues et al., 2025; Shin et al., 2021). These pathophysiological characteristics may contribute to a distinct cholesterol metabolic pattern in comorbid patients, differentiating them from those with aCSVD alone (Tseng et al., 2024; Fallmar et al., 2021).

Imaging analysis revealed significantly higher deep WMH Fazekas scores and DMV scores in the aCSVD-iNPH group compared to the aCSVD group. The aggravated deep WMHs reflect cumulative microcirculatory impairment in white matter and are associated with small venous dysfunction (Chojdak-Lukasiewicz et al., 2021; Dalby et al., 2019). The DMV score is inversely correlated with the visualization of DMVs, and its elevation indicates structural damage and hemodynamic abnormalities in the venous system (Chen et al., 2020; Zhou et al., 2020), as well as impaired venous drainage and perivascular space dysfunction (Zhang et al., 2025; Kang et al., 2022). In iNPH, altered cerebrospinal fluid dynamics increase venous outflow resistance, exacerbating medullary venous congestion and wall fibrosis (Roth et al., 2020; Snobohm et al., 2022; Atasoy et al., 2020); furthermore, the characteristic cerebrospinal fluid circulation disorder in iNPH may worsen deep white matter hypoxia through venous congestion, thereby forming a vicious cycle that promotes WMH progression (Tseng et al., 2024; Snobohm et al., 2022).

The hypothesis proposed by Zhang et al., suggesting that venous system impairment leads to interstitial fluid accumulation, may synergize with the ventricular enlargement mechanism in iNPH (Zhang et al., 2021). This dual dysfunction of the venous and lymphatic systems may be associated with the emergence of deep WMHs, structural damage to DMVs, and hemodynamic abnormalities in aCSVD, potentially contributing to its progression toward iNPH. Recent prospective evidence from patients with aneurysmal subarachnoid hemorrhage has demonstrated that gut dysbiosis—particularly reduced butyrate-producing genera such as Intestinimonas and Butyricimonas, along with enrichment of pro-inflammatory genera such as Acidaminococcus—is closely associated with cerebral vasospasm and delayed cerebral ischemia, highlighting a mechanistic link between gut microbiota, vascular inflammation, and secondary brain injury (Klepinowski et al., 2024). These findings are consistent with the results of the present study, suggesting that similar gut–brain axis pathways may be involved in the pathophysiology of cerebral small vessel disease. On the other hand, gut microbiota dysbiosis may influence venous pathology through the “gut-brain-vascular axis.” This study observed a significant enrichment of Thermus in the aCSVD-iNPH group, which positively correlated with DMV scores. Thermus, belonging to the Deinococcus-Thermus phylum, is a Gram-negative, non-motile rod-shaped bacterium characterized by thermotolerance (45–75 °C) and chemolithoautotrophic capabilities, enabling its participation in metabolic processes such as sulfur cycling (Zhou et al., 2018; Zhou et al., 2020). Its genome contains heat-resistant genes, including heat shock proteins, conferring a survival advantage in extreme environments (Zhou et al., 2018; Zhou et al., 2020). Although traditionally considered an environmental bacterium, its detection in the gut suggests that colonization is potentially influenced by host body temperature, environmental conditions, or disease status (Zhou et al., 2020; Wu et al., 2022; Zhu et al., 2024; Ducray et al., 2019). The possibility of external contamination cannot be excluded. Given the cross-sectional design of this study, these observational findings should be interpreted as exploratory and hypothesis-generating, and their biological significance and underlying mechanisms warrant further investigation.

This study identified a negative correlation between Solobacterium and the presence of basal ganglia and deep CMBs. As a Gram-positive anaerobic bacterium, its role in the gut–brain axis remains unclear. Current evidence suggests it may inhibit microbleed formation through the following mechanisms: Firstly, it may enhance blood–brain barrier integrity through the production of short-chain fatty acids (e.g., butyrate) (Huang J. et al., 2024; Song et al., 2024), which can modulate tight junction protein expression and reduce vascular permeability (Feng et al., 2021). Secondly, it may regulate host immune responses, suppress local inflammatory microenvironments, and mitigate vascular endothelial oxidative stress damage (Huang C. et al., 2024; Shi et al., 2023), aligning with the chronic low-grade inflammation characteristic of aCSVD. Furthermore, this genus may influence methylation metabolism by degrading homocysteine precursors, potentially linking it to hypertension-associated deep CMB formation (Yoo et al., 2020; Graff-Radford et al., 2017). Notably, despite this negative correlation, the number of basal ganglia and deep CMBs in the aCSVD-iNPH group was not significantly lower than in the aCSVD group. This paradoxical observation may stem from: (1) Within the gut microbiota ecological balance, the protective effect of Solobacterium might be counteracted by pro-inflammatory factors such as lipopolysaccharides produced by other enriched genera (e.g., Thermus and Dietzia) in this group (Huang J. et al., 2024; Zhuang et al., 2025), with similar antagonistic effects observed in atherosclerosis models (Baradaran et al., 2023); (2) The unique cerebrospinal fluid dynamics in iNPH may exacerbate perivascular space enlargement through mechanical traction (Duperron et al., 2023), undermining the local protective effects of specific bacterial taxa; and (3) Spatial distribution differences in microbial metabolites may exist, with deep microvascular beds potentially being more susceptible to systemic inflammatory factors than local metabolites (Graff-Radford et al., 2017; Naganuma and Takemoto, 2018). Compared with previous studies, our findings are consistent with Huang et al.’s report on gut microbiota dysbiosis in aCSVD (Huang J. et al., 2024), but differ from the negative correlation pattern between butyrate-producing bacteria and white matter hyperintensity reported by Song et al. (2024). This regional specificity suggests that basal ganglia and deep CMBs may be more influenced by specific bacterial metabolic pathways, whereas cortical CMBs are more associated with microbiota linked to amyloid angiopathy (Pasi et al., 2019; Das et al., 2023).

Finally, this study found that in the aCSVD-iNPH group, the abundance of probiotic-related genera (e.g., *Eubacterium hallii* and Blautia) was significantly reduced, while the relative abundance of opportunistic pathogens (e.g., *Clostridium innocuum* group) was increased. This suggests that gut microbiota dysbiosis may exacerbate neuroinflammation and vascular injury through the gut–brain axis. Existing studies have confirmed the potential value of probiotic interventions in neurodegenerative diseases. For instance, Bifidobacterium can alleviate neuroinflammation by modulating short-chain fatty acid metabolism (Su et al., 2022), while *Clostridium butyricum* has demonstrated cognitive-improving effects in Alzheimer’s disease models (Huang et al., 2021)—the above evidence is still at an exploratory stage. Based on the microbial characteristics identified in this study, it is speculated that targeted modulation of Solobacterium may represent a hypothetical strategy worth future investigation.

This study has the following limitations: (1) The sample size was relatively small, particularly with only 20 cases in the aCSVD-iNPH group, which may affect statistical power; (2) The cross-sectional design cannot establish a causal relationship between gut microbiota alterations and disease progression; (3) The absence of integrated metabolomics or immune marker data limits the depth of mechanistic interpretation. Future research should use longitudinal cohorts combined with multi-omics approaches, quantitative real-time PCR (qPCR) for targeted amplification of specific bacterial taxa, and the establishment of animal models to further analyze and validate the functions of particular genera (such as Solobacterium and Thermus) and their specific pathway associations with neuroinflammation and blood–brain barrier injury. Furthermore, integrating machine learning to develop microbiota-imaging predictive models could optimize early diagnosis and prognostic assessment for aCSVD-iNPH.(4)Several factors known to influence gut microbiota composition—including recent antibiotic use, probiotics, proton pump inhibitors, laxatives, constipation, dietary habits, smoking and alcohol consumption, stool collection conditions, and other medications—were not systematically collected in this study. Additionally, the healthy control group was intentionally selected to be free of hypertension, diabetes, dyslipidemia, and smoking history to serve as a relatively homogeneous reference, which resulted in notable differences in baseline characteristics between the control and patient groups and may have affected the interpretation of microbiota differences. Future studies with more comprehensive data collection, including detailed assessments of dietary habits, medication use, and other potential confounders, as well as more closely matched control groups, are warranted to validate and extend our findings.

## 5 Conclusion

This study reveals distinct alterations in the gut microbiota structure of patients with aCSVD-iNPH, with the microbial composition significantly differing from that of aCSVD patients and HCs. Among these, nine genera—Clostridium_Innocuum_group, Dietzia, Lachnospira, Pedobacter, Romboutsia, Saccharomyces, Solobacterium, Thermus, and TM7x—were significantly enriched in aCSVD-iNPH patients. Further analysis showed that Solobacterium was negatively correlated with basal ganglia and deep CMBs, while Thermus was positively associated with the DMV score. Specific gut microbial taxa may serve as potential diagnostic biomarkers for aCSVD-iNPH, although these preliminary results warrant further validation in independent cohorts.

## Data Availability

The original contributions presented in the study are included in the article/Supplementary material, further inquiries can be directed to the corresponding authors.
