# Correction: Association between gut microbiota and imaging biomarkers in arteriosclerotic cerebral small vessel disease with idiopathic normal pressure hydrocephalus

**DOI:** 10.3389/fnins.2026.1902154

**Published:** 2026-07-31

**Authors:** Jiaxin Cai, Kaiyan Feng, Jiaxin Chen, Lijuan Liang, Qiaoling Wu, Qishan He, Xiong Zhang, Jianming Lei, Jianhui Mai, Yizhong Li, Jinwen Feng, Chunnian Huang, Guofang Zeng, Chunwan Chen, Haifeng Huang, Shisheng Ye, Hao Li

**Affiliations:** 1 The First School of Clinical Medicine, Southern Medical University, Guangzhou, China; 2 Department of Neurology, Maoming People's Hospital, Maoming, China; 3 The First School of Clinical College of Medicine, Guangdong Medical University, Zhanjiang, China; 4 Dongguan Chang'an Hospital, Dongguang, China; 5 Leliu Hospital Affiliated with Shunde Hospital of Guangzhou University of Chinese Medicine, Foshan, China; 6 Department of Integrated Therapy, Maoming People's Hospital, Maoming, China; 7 Department of Radiology, Maoming People's Hospital, Maoming, China; 8 Jinan University, Guangzhou, China

**Keywords:** arteriosclerotic cerebral small vessel disease, gut microbiota, idiopathic normal pressure hydrocephalus, imaging biomarker, Solobacterium

An incorrect number was provided for the Maoming Science and Technology Program Project. The correct number is [2021087].

The original version of this article has been updated.

