# Supplementary material for: Association between gut microbiota and imaging biomarkers in arteriosclerotic cerebral small vessel disease with idiopathic normal pressure hydrocephalus
Source: Front Neurosci. 2026 May 8;20:1789687. doi: 10.3389/fnins.2026.1789687 (PMC13200555; doi:10.3389/fnins.2026.1789687)
Supplement: Supplementary file 1 [file Data_Sheet_1.DOCX]

Table S1 Bacterial Genera Significantly Enriched and Depleted in aCSVD-iNPH Compared to aCSVD

| Enriched | Clostridium innocuum group、Dietzia、Lachnospira、Agathobacter 、Romboutsia、Saccharomyces 、Solobacterium、Thermus、TM7x |
| --- | --- |
| Depleted | Eubacterium hallii、Ruminococcus torques group、Blautia、Clostridia_UCG_014、Clostridium_sensu_srticto_1、Dorea、Escherichia-Shigella、Hungatella、Incertae_Sedis、Parabacteroides、UCG_009 |


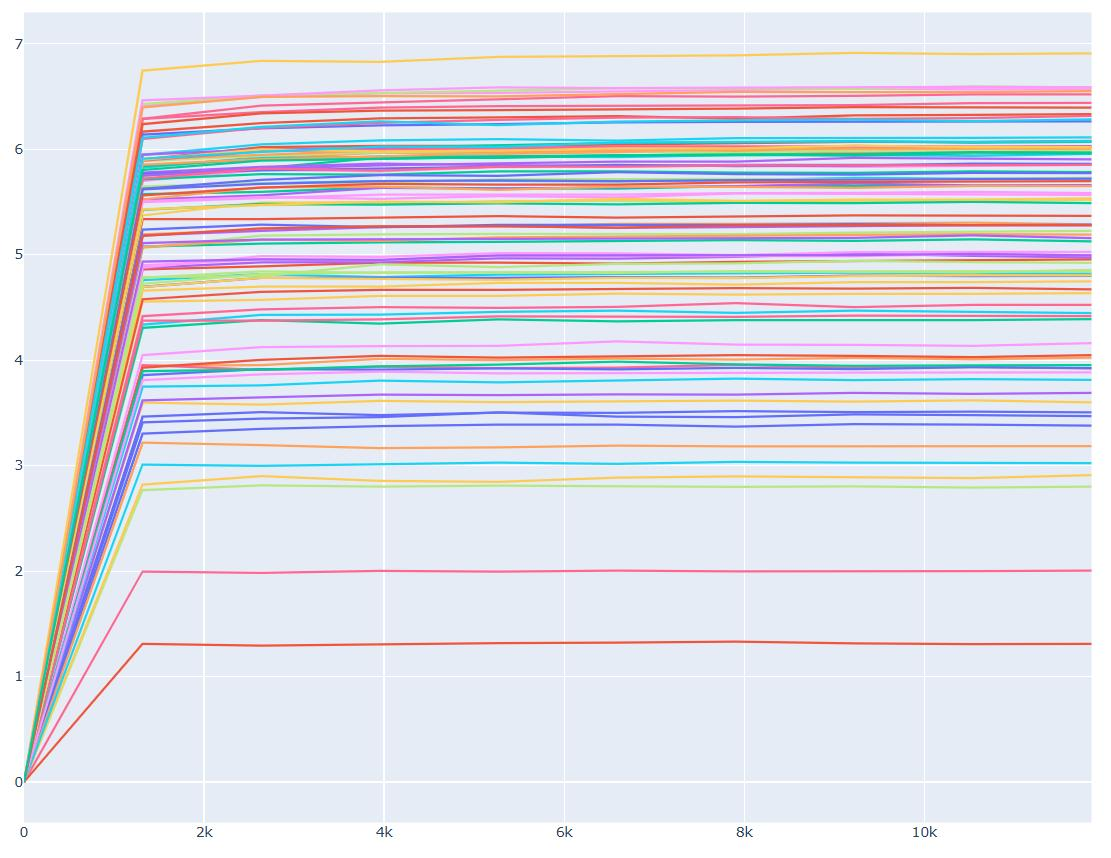


Figure1 Rarefractioncurve


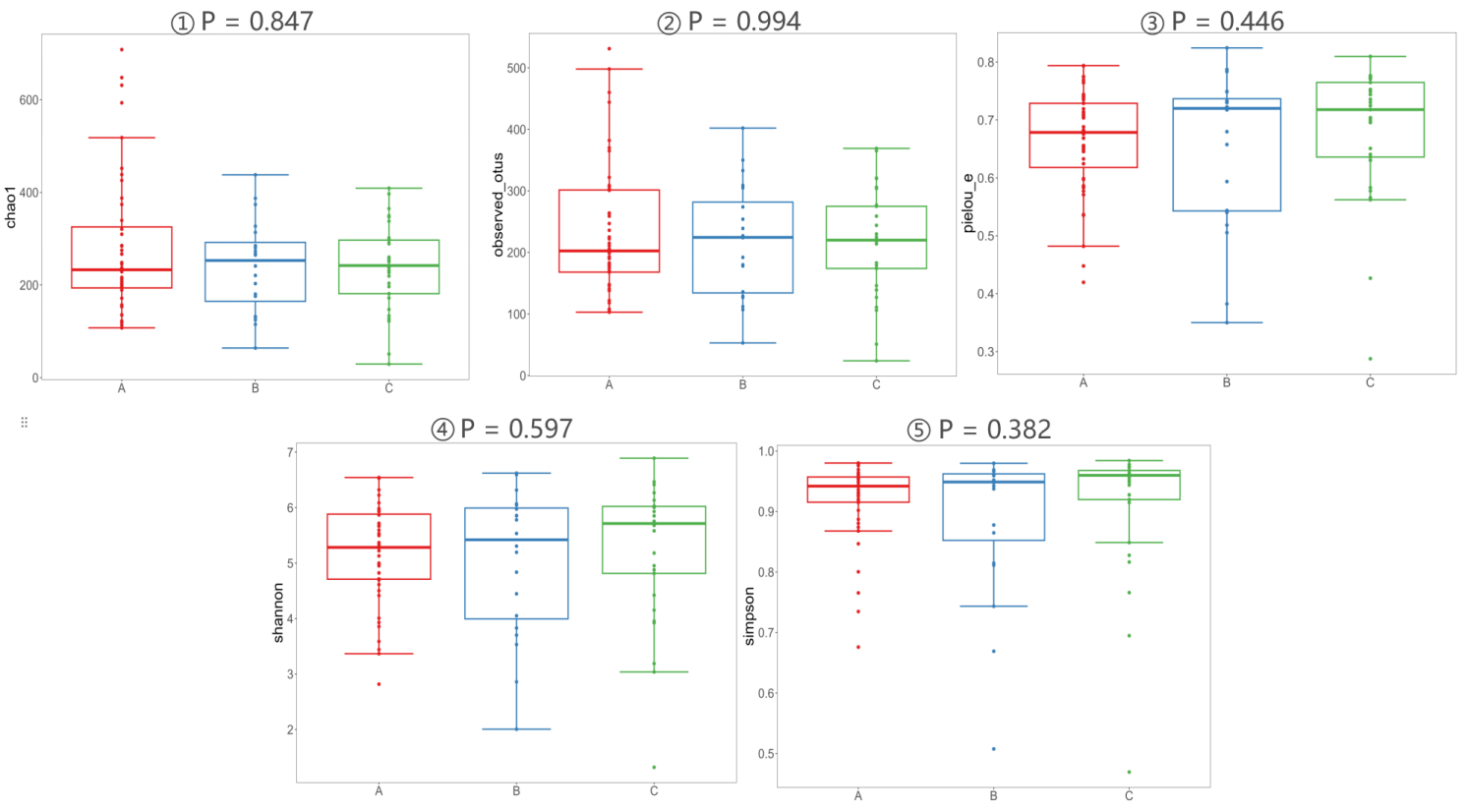


Figure2 The alpha-diversity indices among three groups

A: aCSVD group; B: aCSVD-iNPH group; C: HC group


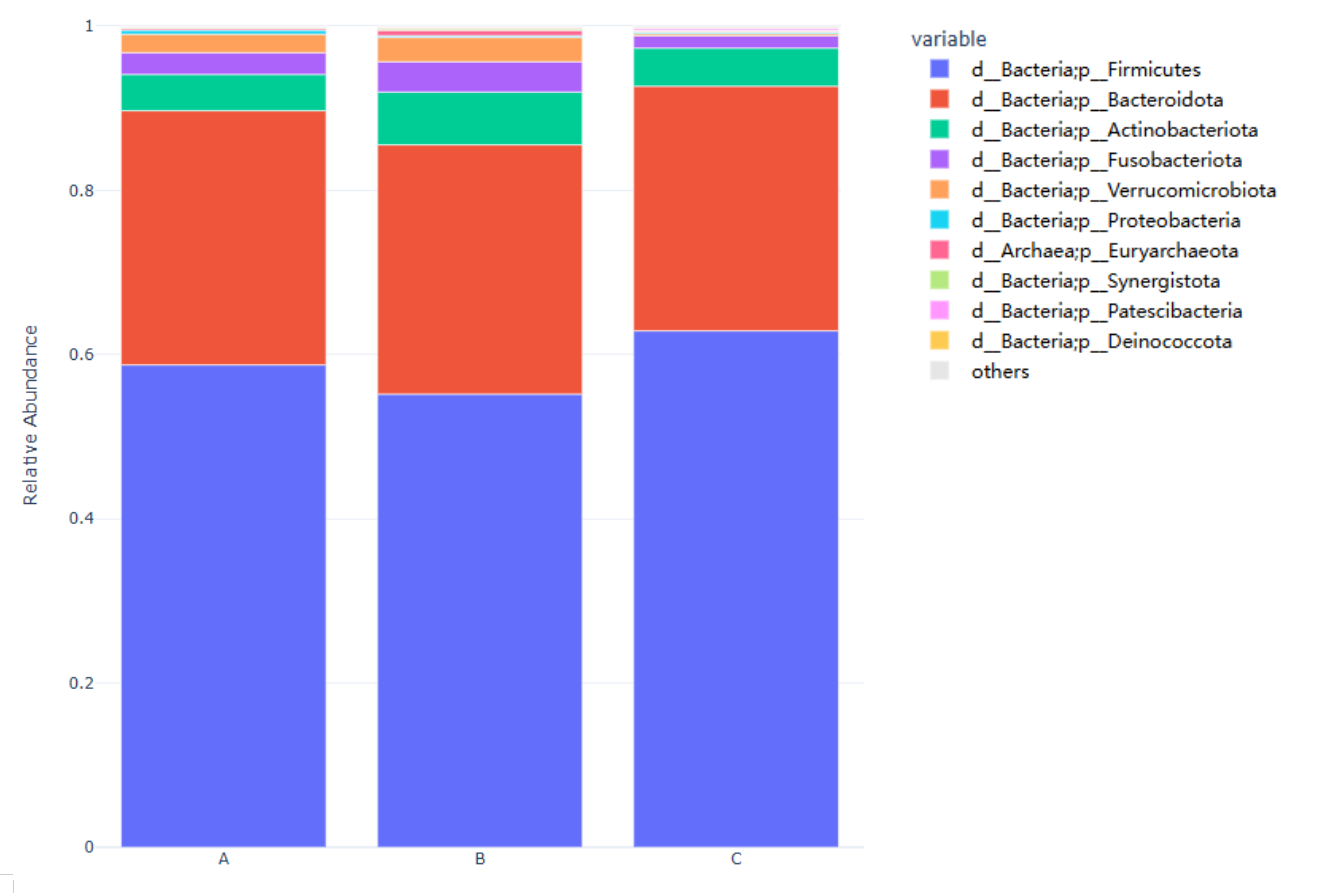


Figure3 Phylum-level relative abundance stacked plot among three groups

A: aCSVD group; B: aCSVD-iNPH group; C: HC group


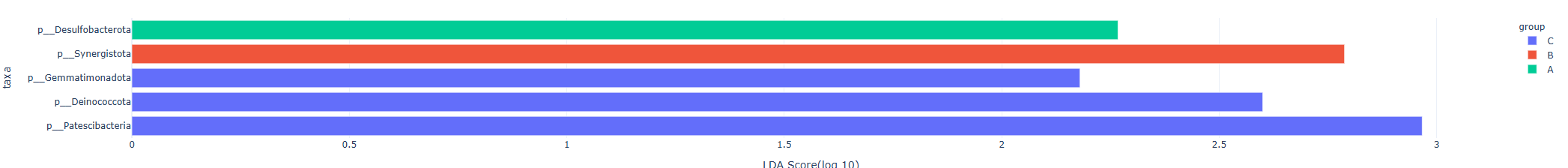


Figure 4 Phylum-level LEfSe analysis

A: aCSVD group; B: aCSVD-iNPH group; C: HC group


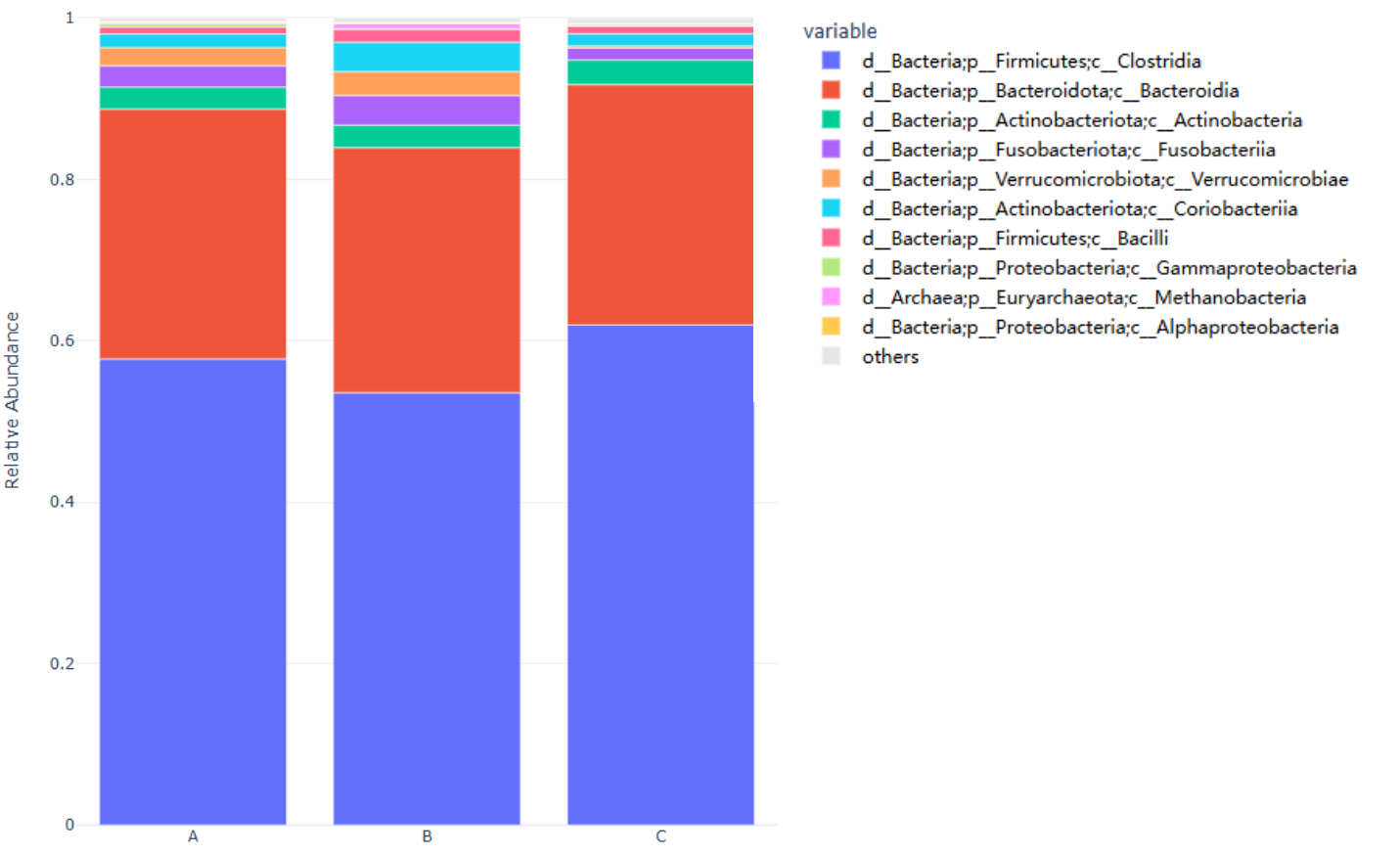


Figure5 Class-level relative abundance stacked plot among three groups

A: aCSVD group; B: aCSVD-iNPH group; C: HC group


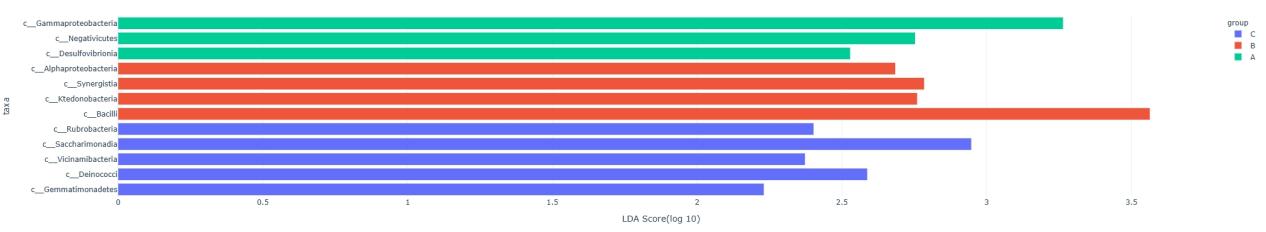


Figure 6 Class-level LEfSe analysis

A: aCSVD group; B: aCSVD-iNPH group; C: HC group


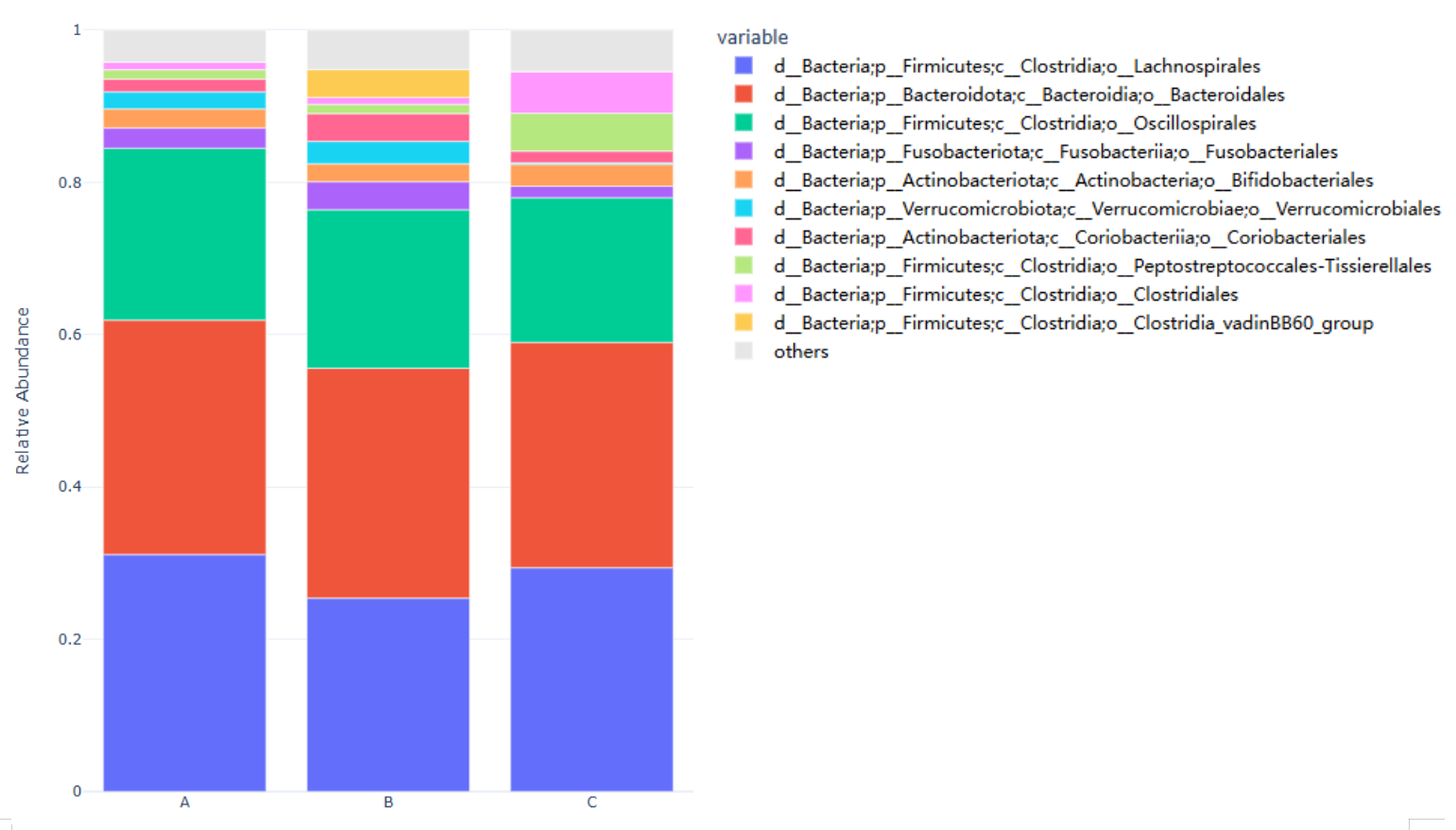


Figure 7 Order-level relative abundance stacked plot among three groups

A: aCSVD group; B: aCSVD-iNPH group; C: HC group


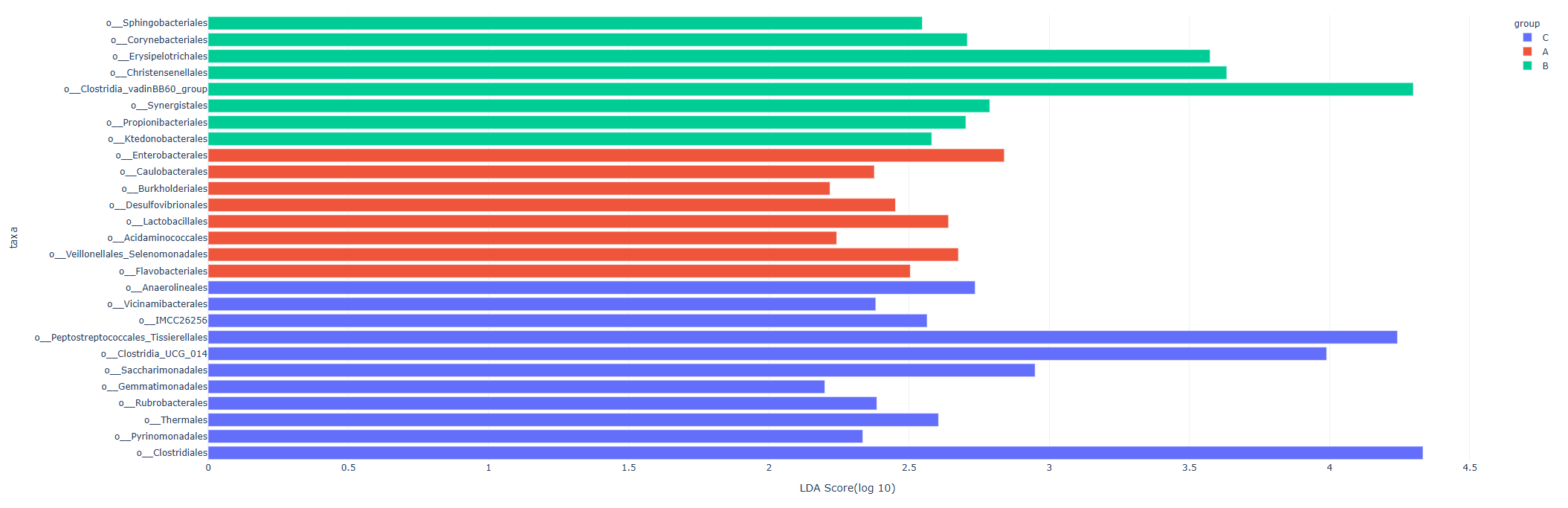


Figure 8 Order-level LEfSe analysis

A: aCSVD group; B: aCSVD-iNPH group; C: HC group


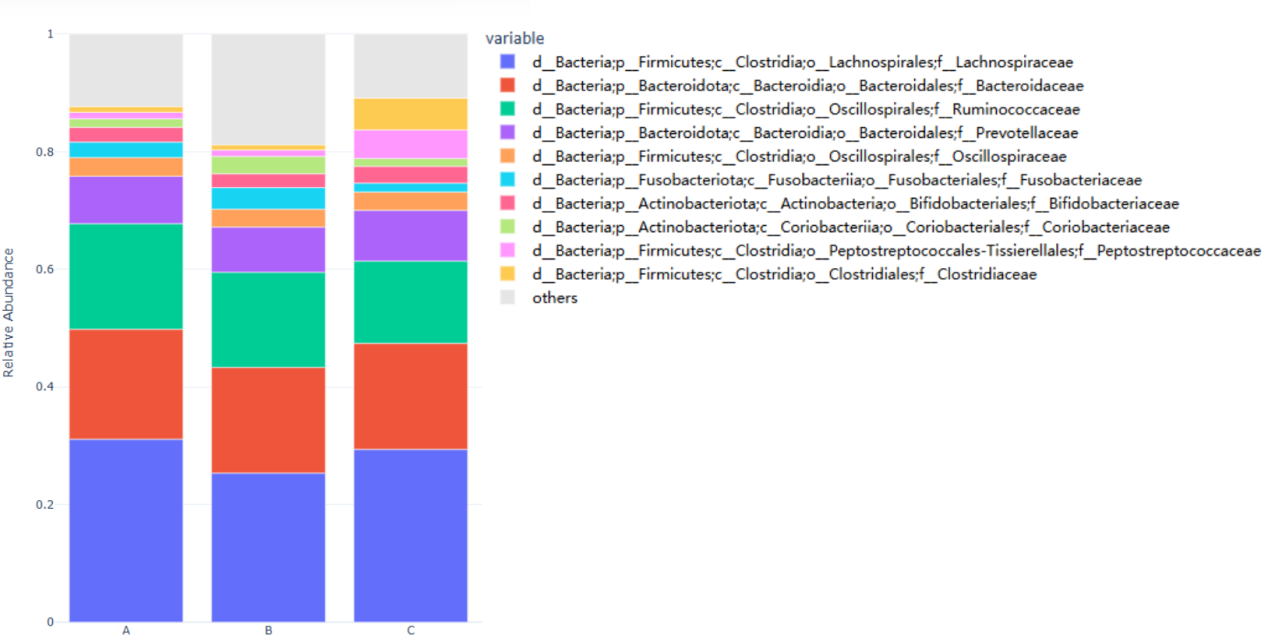


Figure 9 Family-level relative abundance stacked plot among three groups

A: aCSVD group; B: aCSVD-iNPH group; C: HC group


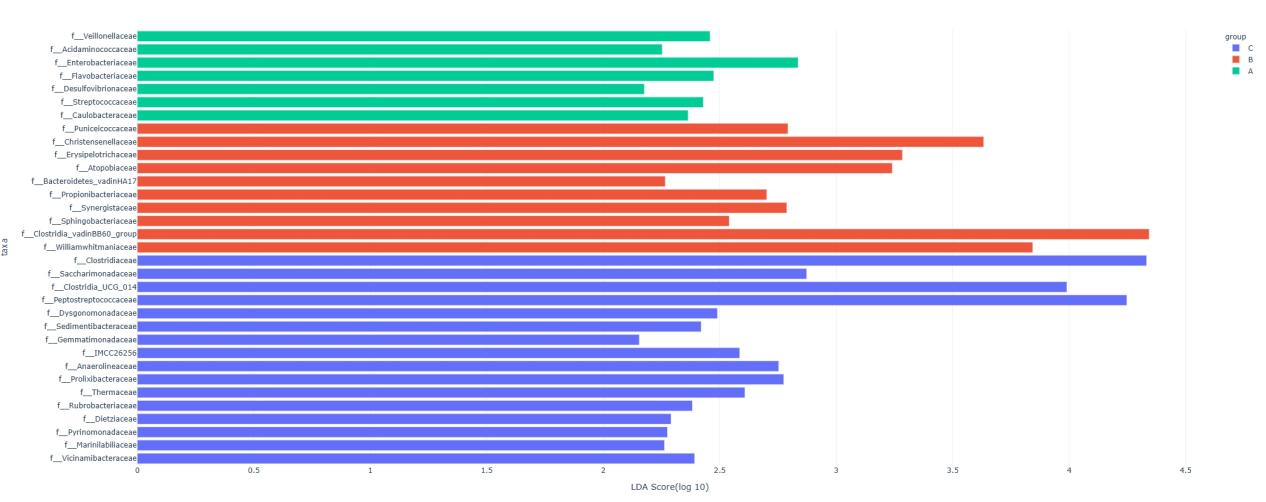


Figure 10 Family-level LEfSe analysis

A: aCSVD group; B: aCSVD-iNPH group; C: HC group


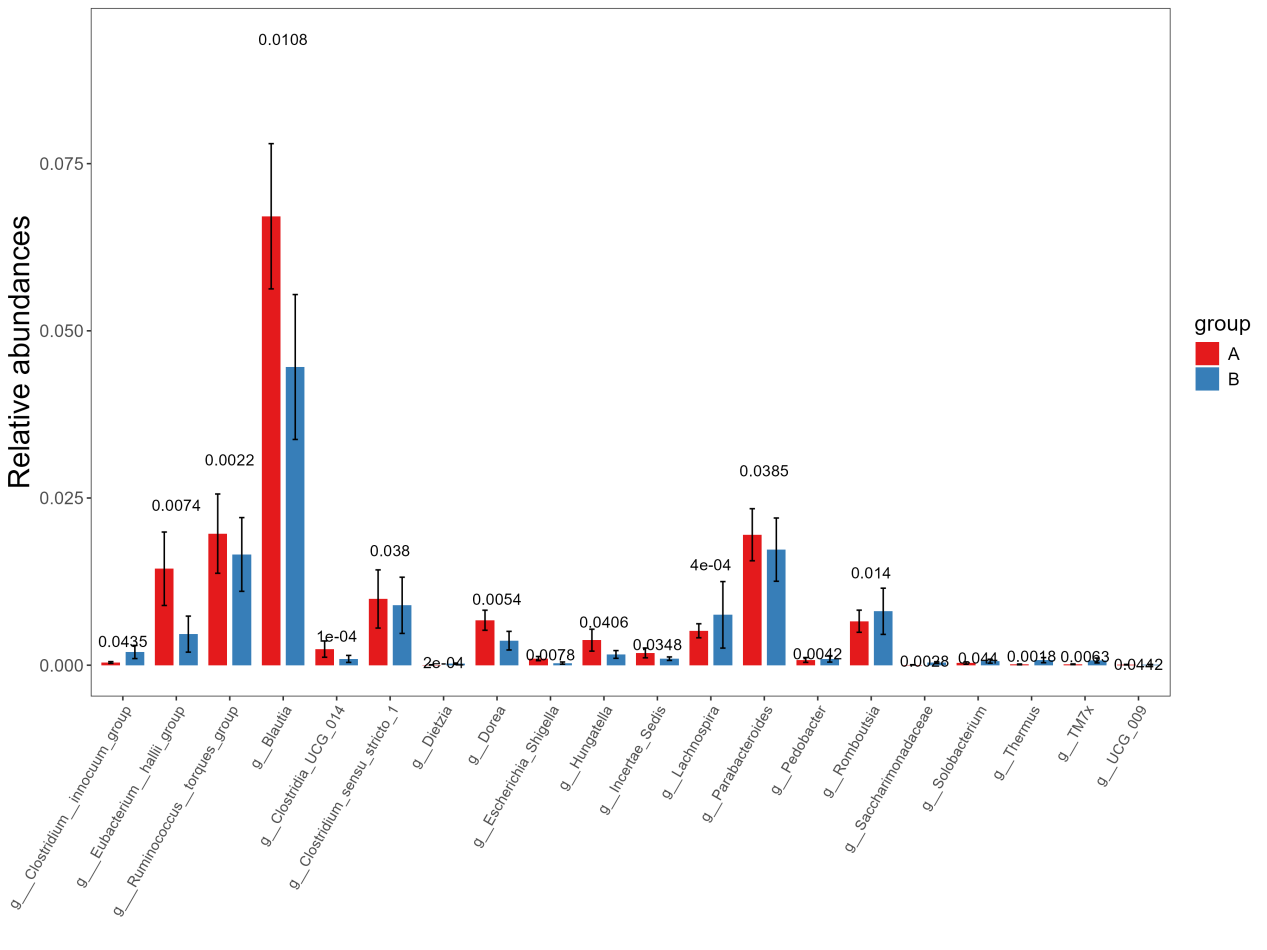


Figure 11Genus-level LEfSe relative abundance among two groups

A: aCSVD group; B: aCSVD-iNPH group
